# *Lactiplantibacillus plantarum* TO-A Reduces Fat Accumulation in *Caenorhabditis elegans* via *pept-1*

**DOI:** 10.3390/microorganisms14030522

**Published:** 2026-02-24

**Authors:** Ryuichi Saito, Rika Inomata, Dian-Sheng Wang, Satoshi Shimazaki

**Affiliations:** Research Division, TOA Biopharma Co., Ltd., 606 Kondoh-cho, Tatebayashi 374-0042, Gunma, Japan; r.inomata@toabio.co.jp (R.I.); dwang@toabio.co.jp (D.-S.W.); shima@toabio.co.jp (S.S.)

**Keywords:** *Lactiplantibacillus plantarum*, probiotics, obesity, *Caenorhabditis elegans*

## Abstract

Lactic acid bacteria (LAB) have dominated food fermentation globally and are ingrained in many food cultures. Obesity is a global health concern, and LAB ingestion is known to exert anti-obesity effects in animals. However, the characteristics of individual bacterial strains and their underlying mechanisms require elucidation since the anti-obesity effects can differ with variations in the strain, host, and living environment. In this study, we aimed to evaluate the safety and anti-obesity effects of *Lactiplantibacillus plantarum* TO-A (LPTOA), isolated from silage, using *Caenorhabditis elegans* as the model organism. The study findings revealed that LPTOA was non-toxic to mice, as established via subacute toxicity tests, and extended the lifespan of *C. elegans*. Furthermore, both LPTOA and heat-killed LPTOA reduced fat accumulation in *C. elegans* by 60% and 58%, respectively. However, in vitro experiments suggested that LPTOA does not decompose cholesterol and triglycerides, nor does it inhibit lipase activity. We identified that *pept-1* (a dipeptide transporter) in *C. elegans* is involved in the anti-obesity effects of LPTOA. PEPT-1 is a protein that controls proton influx into the intestinal tract and is involved in not only peptide uptake but also free fatty acid absorption. These results demonstrate the anti-obesity effects and probiotic potential of LPTOA for application in products, including foods and supplements.

## 1. Introduction

Unhealthy lifestyle habits such as unbalanced diet, lack of physical activity, smoking, alcohol consumption, and stress have led to an increase in the obese population; according to the World Health Organization (WHO), one in eight people worldwide were living with obesity in 2022 [1]. Obesity increases the risk of developing noncommunicable diseases (NCDs), such as diabetes, hypertension, coronary heart disease, stroke, and cancer [2,3]. Therefore, the prevalence of NCDs has also increased with the rise in the obese population [4]. Obesity is a metabolic disease which develops as a result of complex interactions between genetic, socioeconomic, cultural, and psychological factors [3]. Maintaining a balance between energy intake and expenditure over the long term is crucial for addressing obesity; however, this is challenging considering the multifaceted factors contributing to the development of obesity. Consequently, public interest in functional foods and fat-reducing supplements is growing and these markets continue to expand.

The association between obesity and gut microbiota has been known for more than a decade, and several lactic acid bacteria (LAB) strains beneficial in preventing and treating obesity have also been identified [5,6,7,8,9]. However, the response to bacterial administration varies among individuals [10,11,12]. The existence of non-responders is attributed to individual differences in lifestyle habits and gut microbiota composition. Further identifying LAB strains that can improve clinical laboratory values in overweight and obese patients can yield LAB strains tailored to individual health conditions and living environments. Therefore, there is an urgent need to further explore the anti-obesity effects of LAB strains and elucidate their mechanisms of action.

*Caenorhabditis elegans* is a simple animal model with an intestinal tract consisting of 20 epithelial cells [13]. Furthermore, the intestinal environment of *C. elegans* is relatively easy to manipulate relative to that of other animal models, since they can be maintained and passaged in a laboratory environment with *Escherichia coli* OP50 (OP50) as their sole food source. Therefore, *C. elegans* is widely used as a model to explore the beneficial properties of bacteria [14,15]. Because the body of *C. elegans* is transparent, the distribution of lipid droplets and proteins within them can be easily observed without dissection. Furthermore, the use of fluorescently labeled dipeptides and fatty acids enables visualization of dipeptide transport and lipid droplet dynamics in vivo [16,17]. In addition, more than 70% of the *C. elegans* genes related to lipid metabolism are orthologous to human genes [18]; moreover, lipids, such as triglycerides, phospholipids, sphingolipids, and sterols, which are found in human lipid droplets, are also present in *C. elegans* lipid droplets [19]. Therefore, *C. elegans* is the preferred animal model for obesity research.

In our previous study, we showed that a LAB strain, *Lactiplantibacillus plantarum* TO-A (LPTOA), isolated from silage, exhibits high lactic acid production, inhibits the growth of several pathogenic bacteria, and confers resistance to *Staphylococcus aureus* in *C. elegans* [20]. In addition, probiotic interventions containing *L. plantarum* potentially reduce the body mass index, body weight, and waist-to-hip ratio in overweight and obese patients [10]. Although LPTOA has the potential to exhibit anti-obesity effects similar to those of other LAB strains, its safety and anti-obesity effects remain to be evaluated. Hence, in this study, we aimed to evaluate the safety of LPTOA and its effects on fat accumulation in *C. elegans*, and to elucidate some of the mechanisms underlying its anti-obesity effects.

## 2. Materials and Methods

### 2.1. Subacute Oral Toxicity Test

#### 2.1.1. Mouse Experimental Conditions

Five-week-old Institute for Cancer Research (ICR) mice were used to assess subacute oral toxicity. Only healthy animals were used following a 7-day quarantine and acclimatization period. Twenty male and twenty female mice were numbered based on body weight and randomly assigned to groups. After that, it was confirmed that the mean body weight did not differ substantially among the groups. Throughout the quarantine, acclimation, observation, and dosing periods, five animals were maintained in stainless steel wiremesh cages (175 W × 240 L × 145 H mm). The animal rooms were maintained at a temperature of 23 ± 3 °C, relative humidity of 50 ± 10%, air ventilation frequency of 10–20 times/h, light intensity of 150–300 Lux, and a 12-h light/dark cycle. Pelleted food for the experimental animals was purchased from PMI Nutritional International, Inc. (St. Paul, MN, USA). UV-irradiated municipal tap water was provided ad libitum. The experiments were conducted at the Korea Research Institute of Chemical Technology (KRICT; Daejeon, Republic of Korea) and approved by the Association for Assessment and Accreditation of Laboratory Animal Care (AAALAC) International. All mice used in this study were cared for in accordance with the principles outlined in the Guide for the Care and Use of Laboratory Animals by the National Research Council (NRC). All procedures were approved by the Animal Care and Use Committee of KRICT (Approval Code: J-035, Approval Date: 10 October 2002).

#### 2.1.2. Intervention Method

LPTOA powder containing 3.7 × 10^10^ cells/g (TOA Biopharma Co., Ltd., Tokyo, Japan) was suspended in isotonic sodium chloride solution (saline) at a concentration of 10% (*w*/*v*). The control group was administered only saline. Mice fasted for 3 h before administration, and the test substance was administered orally by gavage to each animal divided in two equal doses at a 2-h interval. The dose was calculated according to the fasted body weight (6000 mg/kg) on the day of administration.

#### 2.1.3. Observation and Measurement

Clinical signs and mortality were monitored hourly between one and six hours after administration and then daily thereafter up to day 14. The individual body weights of the animals were measured immediately before administration and on days 1, 3, 7, and 14 after administration. On day 14 after administration, all animals were euthanized by carbon dioxide overdose, and necropsy was performed on all organs and tissues.

### 2.2. C. elegans Strains and Maintenance Conditions

The following *C. elegans* strains were used in the study: N2 Bristol, *lipl-1* (tm1954), *lipl-2* (tm4324), *lipl-4* (tm4417), and *pept-1* (lg601). N2 Bristol and *pept-1* (lg601) were provided by the *Caenorhabditis* Genetics Center (CGC; Minneapolis, MN, USA). *lipl-1* (tm1954), *lipl-2* (tm4324), and *lipl-4* (tm4417) were provided by National Bioresource Project for the Experimental Animal “Nematode *C. elegans*” (NBRP *C. elegans*). The N2 Bristol strain was used as the wild-type. All strains were maintained at 20 °C on the *E. coli* OP50 lawn in nematode growth medium (NGM) agar plates (3 g NaCl, 17 g of agar, 2.5 g peptone, 25 mM KPO_4_ buffer, 1 mM MgSO_4_, 1 mM CaCl_2_, 5 µg/mL cholesterol in ethanol, and 975 mL H_2_O). For all experiments, young adult worms were collected in M9 buffer (3 g KH_2_PO_4_, 6 g Na_2_HPO_4_, 5 g NaCl, 0.02% gelatin, 1 mM MgSO_4_, and 1 L H_2_O) and washed three times before use. To obtain young adult worms, eggs were grown for 3–5 days.

### 2.3. Bacterial Strains and Culture Conditions

The following bacterial strains were used in this study: OP50, LPTOA, *L. plantarum* ATCC14917, and *Lacticaseibacillus rhamnosus* GG (ATCC53103). OP50 was grown at 37 °C in 5 mL Luria–Bertani broth (1 g tryptone, 0.5 g yeast extract, 0.5 g NaCl, and 100 mL H_2_O [pH 7.0]) with shaking for 24 h. LPTOA, *L. plantarum* ATCC14917, and *L. rhamnosus* GG were grown at 37 °C in 5 mL of *Lactobacillus* medium based on the formulation of de Man, Rogosa and Sharpe (MRS) broth without shaking under anaerobic conditions for 24 h. Heat-killed LPTOA (HK_LPTOA) was prepared by heat treating a 10 µg/µL LPTOA suspension (prepared in M9 buffer) at 80 °C for 90 min. Unless otherwise mentioned, in all experiments, 200 µL bacterial suspension (10 µg/µL bacteria in M9 buffer) was spread over the entire surface of 60 mm modified NGM without peptone (mNGM) plates and provided to the worms.

### 2.4. Life Span Assay

*C. elegans* (10–20 in number) were transferred to a bacterial lawn on mNGM. The worms were maintained at 20 °C and transferred to fresh plates (bacterial lawn) every alternate day. A worm was considered dead if it did not exhibit pharyngeal pumping or react with the picker. Worms that died as a result of internal hatching or climbing the plate wall were removed from the plate and not included in the assay.

### 2.5. Oil Red O Staining and Lipid Quantitation

Oil Red O staining and lipid quantitation of the worms were performed as described previously [21,22,23]. Approximately 100 worms which were fed bacteria for one day were collected and washed with M9 buffer. After removing the supernatant, to permeabilize the cuticle, 100 µL M9 buffer and 100 µL 2× Modified Ruvkun’s Witches Brew (MRWB) (160 mM KCl, 40 mM NaCl, 357.5 mg EGTA, 266.3 mg HEPES, and 50 mL H_2_O [pH 7.4]) were added to the worms. Before use, 0.2% β-mercaptoethanol, 2% para-formaldehyde, 1 mM spermidine, and 0.4 mM spermine were added to 2 × MRWB. The samples were then incubated on a rocking shaker at room temperature for 1 h. After removing the supernatant, the worms were washed with the M9 buffer. To dehydrate the animals, the washed worms were treated with 1 mL 60% isopropanol at room temperature for 15 min. Subsequently, 1 mL of Oil Red O solution was added to the dehydrated worms and incubated on a rocking shaker at room temperature for 24 h. The Oil Red O solution was prepared by dissolving 125 mg Oil Red O in 41.75 mL 60% isopropanol. After washing with M9 buffer, the worms were mounted on a 2% agarose pad on a slide glass and visualized under a stereomicroscope with a camera (WRAYCAM-EL310, WRAYMER, Osaka, Japan). The lipid content in the worms was quantitated using image J (ver. 1.54f). At least 30 worms per group were imaged in at least three separate experiments. To provide a dark background, the images were inverted and separated into RGB images. By setting a threshold, the number of pixels in the stained region can be identified from the blue channels of the RGB image. The same threshold was set for all images to determine the pixel count. The obesity level was quantified by dividing the number of pixels within the stained region by the number of pixels representing the entire worm. The fat accumulation ratio was calculated considering the obesity level of the OP50 fed group as 1.0.

### 2.6. Pumping Rate Assay

A pumping rate assay was performed according to the method described by Inomata et al. [24]. Worms were transferred to the bacteria lawn in mNGM and maintained for one day at 20 °C. The movements of the pharyngeal muscles were recorded using a stereomicroscope equipped with a camera (WRAYCAM-EL310, WRAYMER, Osaka, Japan). The number of pumps by the pharyngeal muscles over 30 s was counted using a video editing software (Windows Movie Maker ver. 9.9.9.5), and the pumping rate per minute was calculated based on these data.

### 2.7. Cholesterol Decomposition Assay

Bacteria (10 mg) and 100 µL 10 mg/mL cholesterol were added to 5 mL M9 buffer and cultured at 37 °C for 24 h with shaking. Cholesterol was collected from the cultured suspension using a modified protocol described by Tomaro-Duchesneau et al. [25]. The culture suspension was added to a mixture of 10 mL ethanol and 5 mL 33% KOH and vortexed for 1 min. The solution was then incubated at 37 °C for 15 min. After cooling to room temperature, 30 mL hexane was added to the suspension and vortexed for 1 min. The upper layer was collected and evaporated. After drying, the samples were mixed with ethanol (1 mL). The cholesterol levels in the samples were determined using LabAssayTM Cholesterol (FUJIFILM Wako Pure Chemical Corporation, Osaka, Japan). The absorbance of cholesterol alone was used as the blank. The cholesterol decomposition rate was calculated using the following formula:

Cholesterol decomposition (%) = ((Absorbance of cholesterol alone − Absorbance of sample with added bacteria)/Absorbance of cholesterol alone) × 100

### 2.8. Triglyceride Decomposition Assay

Bacteria (10 mg) and 50 µL 10 mg/mL tributyrin were added to 5 mL M9 buffer and cultured at 37 °C for 24 h with shaking. Suspensions containing triglycerides from the culture solution were collected using a protocol modified from that of Zhao et al. [26], wherein 10 mL ethanol was added to the 200 µL cultured suspension and vortexed for 30 s. After centrifugation at 4000× *g* for 5 min, 1 mL of the supernatant was collected. Triglyceride levels in the samples were determined using LabAssayTM Triglycerides (FUJIFILM Wako Pure Chemical Corporation). The absorbance of the triglycerides was used as the blank. The triglycerides decomposition rate was calculated using the following formula:

Triglyceride decomposition (%) = ((Absorbance of triglyceride alone − Absorbance of sample with added bacteria)/Absorbance of triglyceride alone) × 100

### 2.9. Lipase Inhibition Assay

The lipase inhibition assay was performed as described by Núñez et al. [27]. Here, 2.5 mg/mL lipase (from porcine pancreas) was added to 0.1 M Tris base buffer (0.1 M Tris [pH 7.0] and 5 mM CaCl_2_), and centrifuged at 2000× *g* for 3 min. After collecting the supernatant, 40 µL the bacterial suspension (100 µg/µL bacteria in M9 buffer) was added to 40 µL of the supernatant on ice. Orlistat was used as a positive control. Similar to the bacterial suspension, 40 µL of 100 µg/µL orlistat (in M9 buffer) was added to 40 µL of the supernatant on ice. The sample was supplemented with 20 µL 10 mM p-Nitrophenyl butyrate (PNPB) (made by 0.1 M Tris base buffer). After mixing by pipetting, the absorbance (400 nm) was measured every 5 min at 37 °C. The absorbance of a sample without PNPB was used as the blank. The lipase inhibition rate was calculated using the following formula:

Lipase inhibition (%) = ((Absorbance of lipase alone − Absorbance of sample with added bacteria)/Absorbance of lipase alone) × 100

### 2.10. RNA Purification and RT-qPCR Analysis

Worms that fed the bacteria for one day at 20 °C were collected in the lysis solution and were immediately frozen in liquid nitrogen [28]. A total of 100 worms were collected per condition for each experiment. The lysis solution consisted of 0.5% Triton X-100, 0.5% Tween 20, 0.25 mM EDTA, 2.5 mM Tris-HCl, pH 8.0, 8% RNAsecure™ RNase Inactivation Reagent (Thermo Fisher Scientific Inc., Waltham, MA, USA), and 1 mg/mL proteinase K. To prepare the worm lysates, these solutions were heated at 65 °C for 15 min, followed by heating at 85 °C for 1 min. To the worm lysates, 100% ethanol (2.5 times volume of sample) and 3 M sodium acetate (10% volume of sample) were added and incubated at −20 °C overnight. After centrifugation at 14,000× *g* for 20 min, RNA was purified from the precipitate of the worm lysates and cDNA was synthesized using the PrimeScript™ RT reagent Kit with gDNA Eraser (Takara Bio Inc., Kyoto, Japan). Reverse transcription quantitative polymerase chain reaction (RT-qPCR) was performed using TB Green^®^ Premix Ex Taq™ II (Takara Bio Inc.) on the Quantstudio 1 real-time PCR System (Thermo Fisher Scientific Inc.). Primers used for RT-qPCR are listed in Table S1. Relative abundances were determined using the delta-delta threshold cycle (Ct) method (2^−ΔΔCt^), and the four housekeeping genes (*tba*-1, *cdc*-42, *eif*-3.c, and *pmp*-3) were used to control the template levels [29].

### 2.11. Statistical Analyses

Statistical analyses were performed using the R software (version 4.4.0). Statistical significance was defined as *p* < 0.05. These values are annotated as ns (non-significant), * *p* < 0.05, and ** *p* < 0.01; letters a, b, and c indicate significant differences between groups (*p* < 0.05).

## 3. Results

### 3.1. Safety Evaluation of LPTOA

**Figure 1:**
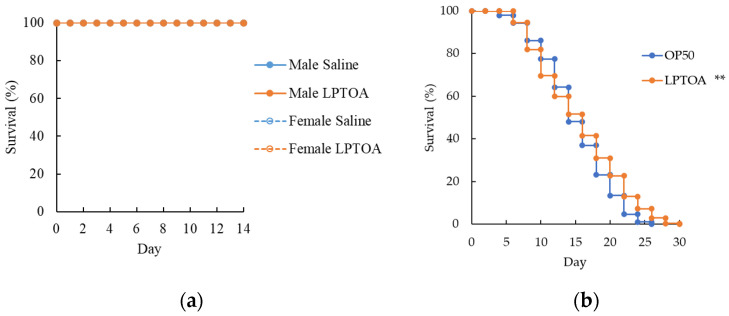
Toxicity test results of LPTOA in mice and worms. (**a**) Subacute oral toxicity test results in mice. *n* = 10. (**b**) Effect of chronic LPTOA ingestion on the lifespan of worms. OP50: *n* = 297, LPTOA: *n* = 277. Significant differences were determined using the log–rank test. ** *p* < 0.01.

Subacute toxicity of LPTOA was evaluated in five-week-old ICR mice following oral administration. Mortality was not observed in either the LPTOA or saline groups during the test period (Figure 1a). Additionally, body weight changes in the LPTOA group were similar to those in the saline group during the test period; gross organ abnormalities were not observed in the LPTOA group following necropsy (Table S2). Consequently, LPTOA was considered to be safe for oral administration in mice at doses up to 6000 mg/kg, and its oral LD50 was estimated to be greater than 6000 mg/kg. Next, we investigated the effects of LPTOA on the lifespan of *C. elegans*. The lifespan of LPTOA-fed worms was significantly longer than that of OP50-fed worms (Figure 1b). These results suggest that LPTOA did not induce serious adverse health effects in mice or worms.

### 3.2. LPTOA Reduces Fat Accumulation in C. elegans

**Figure 2:**
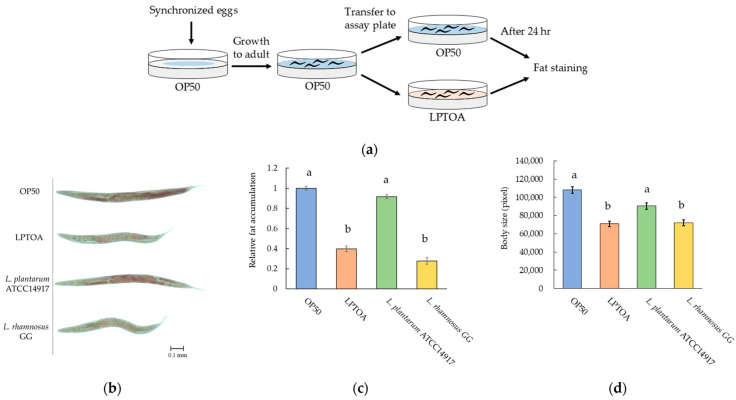

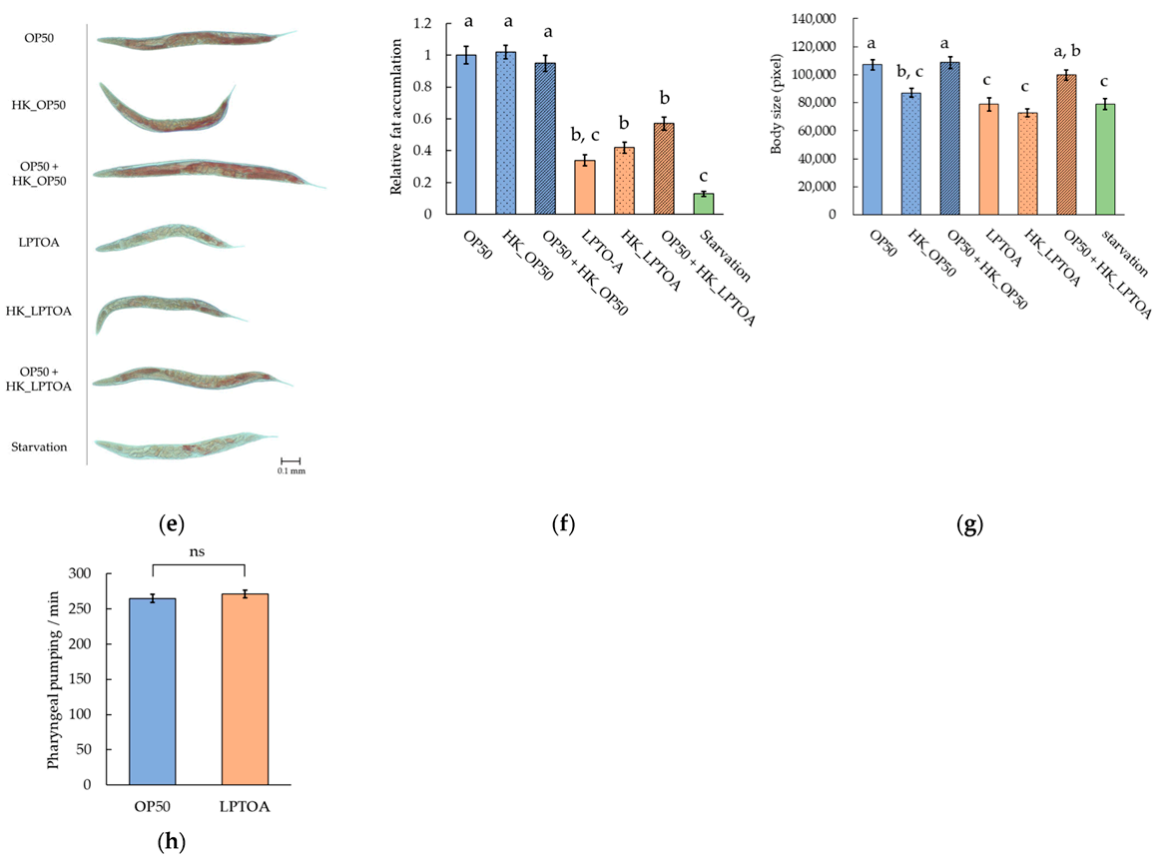
Evaluation of the ability of LAB to reduce fat accumulation in *C. elegans*. (**a**) Experimental procedure for evaluating fat accumulation in worms. (**b**) Representative images of worms stained with Oil Red O after being fed OP50, the three LAB strains, OP50 + HK_OP50: mixture of OP50 and HK_OP50, or OP50 + HK_LPTOA: mixture of OP50 and HK_LPTOA. (**c**) Relative quantification of Oil Red O-stained areas corresponding to (**b**). *n* = 30. (**d**) Body size measurements (number of pixel) of the worms corresponding to (**b**). *n* = 30. (**e**) Representative images of worms stained with Oil Red O after being fed HK_OP50 or HK_LPTOA. (**f**) Relative quantification of Oil Red O-stained areas corresponding to (**e**). *n* = 30. (**g**) Body size measurements (number of pixel) corresponding to (**e**). *n* = 30. (**h**) Average number of pharyngeal pumps per minute in worms fed OP50 or LPTOA. *n* = 30. (**c**,**d**,**f**,**g**,**h**) Error bars indicate standard errors. Significant differences were determined using Dunn’s test (Holm). Significant differences between groups are indicated by letters (*p* < 0.05). ns: non-significant.

The amount of fat accumulation in worms fed LPTOA, *L. plantarum* ATCC14917, or *L. rhamnosus* GG was evaluated relative to that in OP50-fed worms. Figure 2a presents a schematic presentation of the experimental design. *L. plantarum* ATCC14917 is known to have no effect on fat accumulation in worms [30]. Consistently, we also observed that relative fat accumulation in the *L. plantarum* ATCC14917-fed group (0.92 ± 0.02) was similar to that in the OP50-fed group (Figure 2b,c). In addition, *L. rhamnosus* GG is one of the most widely studied probiotic strains and is known to reduce fat accumulation in worms [30]. Consistently, we observed that relative fat accumulation in the *L. rhamnosus* GG-fed group (0.28 ± 0.03) was significantly lower than that in the OP50-fed group (Figure 2b,c). Meanwhile, relative fat accumulation of the LPTOA-fed group was 0.40 ± 0.03, which was significantly lower than that of the OP50-fed group and was similar to that of the *L. rhamnosus* GG-fed group (Figure 2b,c). Furthermore, the changes in body size of the worms were similar to their relative fat accumulation across the groups, suggesting a correlation between the two (Figure 2d).

Next, we investigated the reduction in fat accumulation in HK_LPTOA-fed worms (Figure 2e,f). The relative fat accumulation values in the heat-killed OP50 (HK_OP50)-fed group and the group fed a mixture of OP50 and HK_OP50 (OP50 + HK_OP50) were 1.02 ± 0.04 and 0.95 ± 0.05, respectively. These values were comparable to those observed in the OP50-fed group. In addition, the relative fat accumulation values of the HK_LPTOA-fed group and the group fed a mixture of OP50 and HK_LPTOA (OP50 + HK_LPTOA) were 0.42 ± 0.03 and 0.57 ± 0.04, respectively. These values were not significantly different from those of the LPTOA-fed group (0.34 ± 0.03). Moreover, relative fat accumulation in the starving group was 0.13 ± 0.02. This value was not significantly different from that observed in the LPTOA-fed group; however, it was significantly lower than those observed in the HK_LPTOA- and OP50 + HK_LPTOA-fed groups. Furthermore, the body size of worms in the OP50-fed group was 107,150 ± 3669 pixels, while those of the HK_OP50-, LPTOA-, and HK_LPTOA-fed groups as well as the starving group were 86,907 ± 3126 pixels, 78,840 ± 4607 pixels, 72,839 ± 2860 pixels, and 79,006 ± 3697 pixels, respectively (Figure 2g). All these values were significantly lower than that of the OP50-fed group. However, the body sizes in the OP50 + HK_OP50- and OP50 + HK_LPTOA-fed groups were 108,597 ± 4004 pixels and 99,821 ± 3716 pixels, respectively (Figure 2g), which were comparable to that of the OP50-fed group. These data indicated that combining HK_OP50 or HK_LPTOA with OP50 ameliorated the decrease in the body size of the worms. Furthermore, these findings suggest that the observed reduction in fat accumulation is not a secondary consequence of growth inhibition. The body size of worms is influenced by the ingested bacteria [31,32]. Reduced food intake leads to decreased body size and fat accumulation in worms [33]. In this study, worms were constantly exposed to bacteria, as the entire surface of the plates was coated with bacteria. However, the feeding frequency of the worms on the plate was unknown. Therefore, we investigated the number of pumping events in the worms (Figure 2h). As a result, the number of pumps in worms on the LPTOA was similar to that in worms on OP50. This suggests that the relative fat accumulation observed in worms fed the LPTOA diet was not caused by a decrease in their feeding frequency.

### 3.3. Lipolytic and Lipase Inhibitory Activity of LPTOA

#### 3.3.1. LPTOA Did Not Exhibit Lipolytic Activity

**Figure 3:**
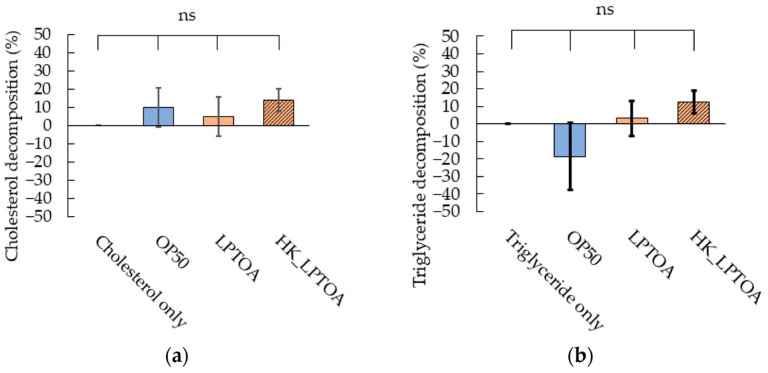
Evaluation of the lipolytic ability of LPTOA. Decomposition rate of cholesterol (**a**) or triglycerides (**b**) when OP50, LPTOA, or HK_LPTOA were cultured for 24 h in M9 buffer containing cholesterol or triglycerides. *n* = 3. (**a**,**b**) Error bars indicate standard errors. Significant differences (*p* > 0.05) were determined using Dunn’s test (Holm). ns: non-significant.

Some *L. plantarum* strains exhibit lipolytic activity and can reduce cholesterol or triglyceride content in the medium [34,35]. Hence, LPTOA was used, owing to its potential to decompose fatty acids in the intestinal lumen of worms. To investigate the lipolytic activity of LPTOA, the bacteria were incubated for 24 h in M9 buffer containing cholesterol or triglycerides (Figure 3a,b). OP50, LPTOA, and HK_LPTOA did not degrade cholesterol or triglycerides.

#### 3.3.2. LPTOA Does Not Possess Lipase Inhibitory Activity

**Figure 4:**
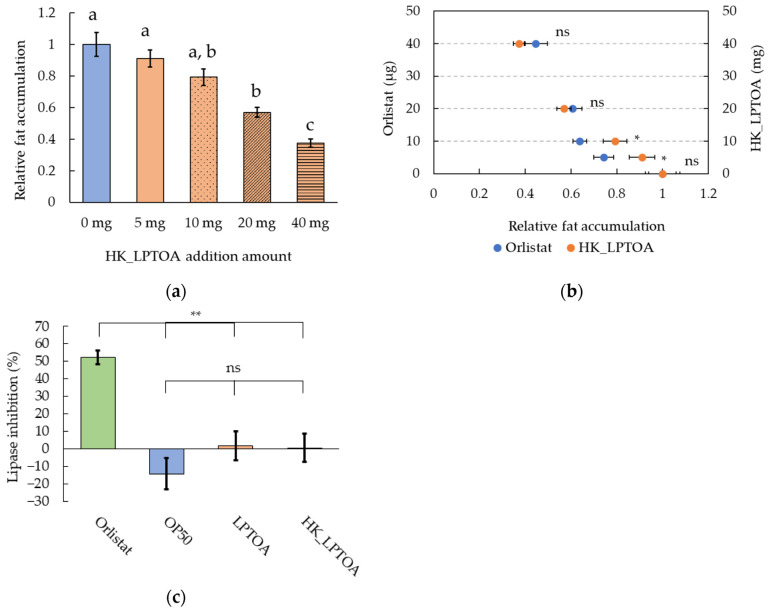
Effects of orlistat and HK_LPTOA on lipase activity and fat accumulation in *C. elegans*. (**a**) Relative quantification of Oil Red O-stained areas in worms fed OP50 supplemented with different amounts of HK_LPTOA. *n* = 30. Significant differences between the groups are indicated by letters (*p* < 0.05). (**b**) Relative quantification of Oil Red O-stained areas in worms fed OP50 supplemented with different amounts of HK_LPTOA or orlistat (referred from panel (**a**)) *n* = 30. Significant differences were determined using Student’s *t*-test. ns: non-significant, * *p* < 0.05. (**c**) Percentage of lipase inhibition by orlistat, OP50, LPTOA, or HK_LPTOA, measured 15 min after substrate addition. *n* = 3. ns: non-significant, ** *p* < 0.01. (**a**,**c**) Significant differences were determined using Dunn’s test (Holm). (**a**–**c**) Error bars indicate standard errors.

Orlistat reduces obesity by inhibiting lipase, thus preventing fat absorption in the intestinal epithelium. Orlistat is known to reduce fat accumulation in *C. elegans* [36,37]. Moreover, some *L. plantarum* strains exhibit lipase inhibitory activities [38]. Hence, similar to orlistat, LPTOA is hypothesized to inhibit lipase activity. When worms were fed a combination of orlistat or HK_LPTOA with OP50, both mixtures reduced fat accumulation in the worms in a concentration-dependent manner (Figure 4a,b). When ≤10 mg HK_LPTOA was fed to the worms, fat accumulation did not reduced significantly; however, administering ≥20 mg HK_LPTOA significantly reduced fat accumulation (Figure 4a). At low concentrations, the orlistat-supplemented group exhibited significantly lower fat accumulation than that of the HK_LPTOA group. The relative fat accumulation values in 5 µg and 10 µg orlistat-added groups were 0.74 ± 0.04 and 0.64 ± 0.03, respectively, while those of the 5 mg and 10 mg HK_LPTOA-added groups were 0.91 ± 0.06 and 0.79 ± 0.05, respectively (Figure 4b). However, at high concentrations, no significant difference was observed between the orlistat- and HK_LPTOA-added groups. The relative fat accumulation values in the 20 µg and 40 µg orlistat-added groups were 0.60 ± 0.04 and 0.45 ± 0.05, respectively, while those in the 20 mg and 40 mg HK_LPTOA-added groups were 0.57 ± 0.03 and 0.37 ± 0.03, respectively (Figure 4b). However, unlike orlistat, OP50, LPTOA, and HK_LPTOA did not inhibit lipase-mediated PNPB degradation (Figure 4c). In addition, lipase activity was not detected in the cells cultured with OP50, LPTOA, or HK_LPTOA alone (Figure S1a). Co-incubation with lipase and OP50 tended to increase lipase activity over time; however, this increase was not significant relative to that on incubation with lipase alone (Figure S1a,b). Furthermore, even after prolonged co-incubation with lipase, LPTOA and HK_LPTOA did not affect lipase activity (Figure S1a,b). Orlistat is a pure compound, whereas HK_LPTOA is a complex. In addition, the concentration of orlistat added was 1000 times lower than that in HK_LPTOA. Consequently, these fundamental differences in composition and dosage limit the extent to which their effects can be directly compared. However, the findings suggested that the fat-limiting effect of HK_LPTOA occurred via a mechanism different from that of orlistat.

### 3.4. Identifying C. elegans Genes Regulating LPTOA-Mediated Reduction in Fat Accumulation

#### 3.4.1. LPTOA Activates *lipl-1* Expression in *C. elegans*

**Figure 5:**
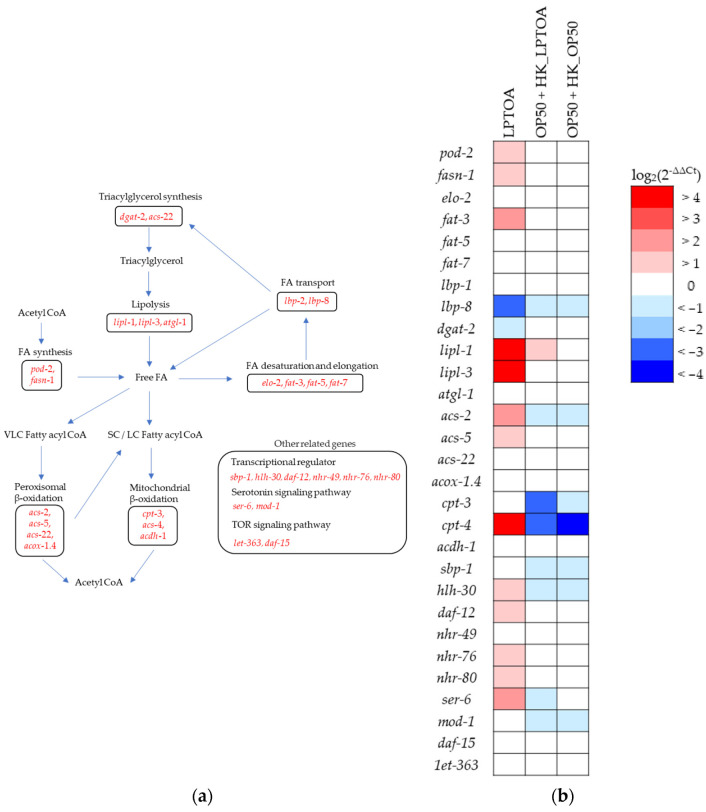
Changes in the expression levels of lipid metabolism-related genes in *C. elegans* following LPTOA or HK_LPTOA intake. (**a**) Schematic diagram of *C. elegans* lipid metabolic pathways involving the selected genes. FA: Fatty acid, SC: short chain, LC: Long chain, VLC: Very long chain. (**b**) heat map comparing gene expression levels in the LPTOA-, OP50 + HK_LPTOA- and OP50 + HK_OP50-fed groups with the OP50-fed group serving as the standard (*n* = 3).

As shown in Figure 3 and Figure 4, LPTOA was incapable of degrading cholesterol or triglycerides, nor could it inhibit lipase. Therefore, we hypothesized that LPTOA reduces fat accumulation in worms by regulating lipid metabolic pathways, including synthesis, storage, and lipolysis. RT-qPCR analysis was performed on 29 lipid metabolism-related genes to assess changes in their expression levels in the LPTOA-, OP50 + HK_LPTOA- and OP50 + HK_OP50-fed groups, with the OP50-fed group serving as the control (Figure 5a and Table S3) [39,40,41,42,43,44,45,46]. LPTOA intake upregulated the expression of several genes in worms by more than two-fold (log_2_ 2^−ΔΔCt^ ≧ 1.0) (Figure 5b). However, many of these genes were not upregulated more than two-fold in the OP50 + HK_LPTOA- and OP50 + HK_OP50-fed groups (Figure 5b). Moreover, the pattern of gene expression in the OP50 + HK_LPTOA-fed group was similar to that in the OP50 + HK_OP50-fed group (Figure 5b). Among the genes analyzed in this study, only *lipl-1* was upregulated by more than two-fold in both the LPTOA- (6.79 ± 0.24) and OP50 + HK_LPTOA-fed (1.90 ± 0.39) groups (Figure 5a and Table S3).

#### 3.4.2. *lipl-1* Is Not Required for LPTOA-Mediated Fat Accumulation Reduction

**Figure 6:**
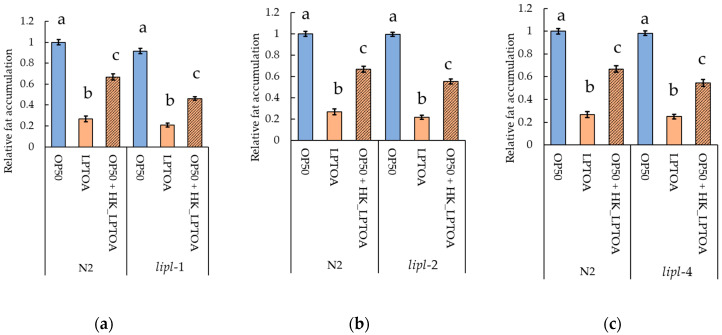
Evaluation of LPTOA-mediated fat accumulation reduction in three lipase gene mutants. Relative quantification of Oil Red O-stained areas in *lipl-1* (**a**), *lipl-2* (**b**), and *lipl-4* (**c**) mutants fed OP50, LPTOA, or OP50 + HK_LPTOA. N2: wild-type. *n* = 30. Error bars indicate standard errors. Significant differences between groups were determined using Dunn’s test (Holm) and are indicated by letters (*p* < 0.05).

To confirm the requirement of *lipl-1*, identified as a candidate gene using RT-qPCR, for LPTOA-mediated fat accumulation reduction, we compared fat accumulation between *lipl-1* mutant (tm1954) and wild-type (N2) (Figure 6a). *C. elegans* possess multiple lipase genes. Hence, other genes belonging to the same family as *lipl-1* may also be involved in reducing LPTOA-mediated fat accumulation. Therefore, fat accumulation in *lipl-2* (tm4324) and *lipl-4* (tm4417) mutants was also compared with that in the wild-type (Figure 6b,c). Consequently, no significant differences in fat accumulation were observed in *lipl-1*, *lipl-2* and *lipl-4* mutants compared to that in the wild-type across all feeding conditions (Figure 6).

#### 3.4.3. *pept-1* Is Involved in LPTOA-Mediated Fat Accumulation Reduction

**Figure 7:**
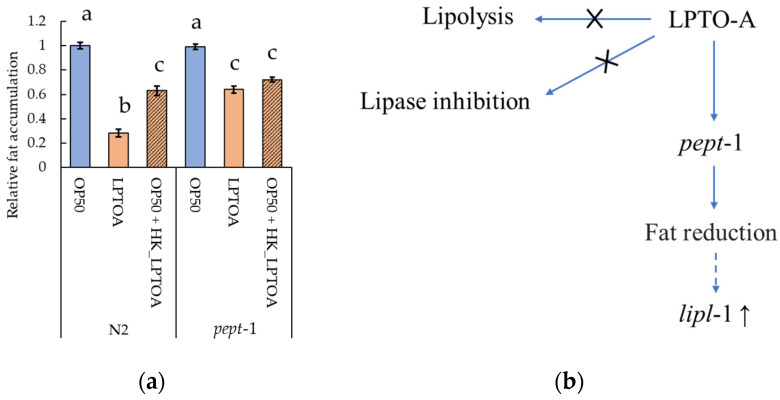
Evaluation of the LPTOA-mediated fat accumulation reduction in *pept-1* mutants and predicted mechanism of action of LPTOA. (**a**) Relative quantification of Oil Red O-stained areas in *pept-1* mutants fed OP50, LPTOA, or OP50 + HK_LPTOA. *n* = 30. Error bars indicate standard errors. Significant differences between groups were determined using Dunn’s test (Holm) and are indicated by letters (*p* < 0.05). (**b**) Schematic diagram for the predicted mechanism of LPTOA-mediated fat accumulation reduction in *C. elegans* in this study. LPTOA does not possess lipolytic or lipase inhibitory activity (indicated by ×); it reduces fat accumulation in *C. elegans* via *pept-1* (indicated by solid arrows). In addition, the upregulation of *lipl-1* expression is not directly controlled by LPTOA but is rather mediated by the reduction in fat accumulation (indicated by dashed arrow).

In addition, we focused on PEPT-1, a transmembrane dipeptide transporter expressed in intestinal epithelial cells. PEPT-1 affects fat accumulation and lipid composition by contribution to the absorption of dietary amino acids and free fatty acids [17,47]. To investigate the relationship between *pept-1* and LPTOA-mediated reduction in fat accumulation, we compared fat accumulation in *pept-1* mutants (lg601) with that in wild-type worms under the following feeding conditions: OP50, LPTOA, or OP50 + HK_LPTOA (Figure 7a). The results revealed that the relative fat accumulation in *pept-1* mutants fed OP50 (0.99 ± 0.02) did not significantly differ from that in wild-type worms fed OP50. In contrast, while LPTOA significantly reduced fat accumulation in wild-type worms (0.28 ± 0.03), this reduction was significantly suppressed in the *pept-1* mutants (0.64 ± 0.03). This result suggests that *pept-1* is involved in the LPTOA-mediated reduction in fat accumulation. However, the relative fat accumulation in LPTOA-fed *pept-1* mutants was similar to those in wild-type worms fed OP50 + HK_LPTOA (0.63 ± 0.04) and *pept-1* mutant fed the same mixture (0.72 ± 0.02). Thus, genes other than *pept-1* are also potentially involved in reducing LPTOA-mediated fat accumulation.

## 4. Discussion

In this study, we revealed that LPTOA reduced fat accumulation in worms, and that this effect was not lost even after heat-killing (Figure 2b,c,e,f). Moreover, HK_LPTOA-mediated fat accumulation reduction was altered based on the HK_LPTOA intake volume (Figure 4a). Thus, some heat-resistant substances in LPTOA reduced fat accumulation in worms. Although *L. plantarum*-induced reduction in fat accumulation in *C. elegans* is well-established, this effect has been poorly investigated in heat-killed *L. plantarum*, with its causative substance being unrecognized [10,30,38,48,49]. To our knowledge, this study is the first to report that both live and heat-killed LPTOA reduce fat accumulation in *C. elegans*. Consequently, to further elucidate the mechanism of action of LPTOA, it is essential to identify the active compound(s) responsible.

Generally, dietary lipids are ground into fine lipid droplets via antral peristalsis and the action of acidic lipases in the stomach. Once they enter the small intestine, these lipid droplets form micelles by interacting with bile acids and are then disassembled into free fatty acids and monoglycerides by lipases. Subsequently, the micelles are transported to the microvilli of intestinal epithelial cells, and the lipid components released from them are absorbed into the cells [50]. Although the pathways of lipid disassembly and absorption are not fully elucidated in *C. elegans*, most lipids stored in worms originate from dietary lipids under laboratory conditions [51]. Furthermore, since orlistat reduces fat accumulation in worms [37], it is plausible that *C. elegans* has lipid digestion and absorption mechanisms similar to those found in mammals. However, since LPTOA and HK_LPTOA did not exhibit lipolytic or lipase inhibitory activity (Figure 3 and Figure 4c), these findings suggest that LPTOA and HK_LPTOA do not affect lipid degradation in the intestinal tract of worms. Hence, we hypothesized that LPTOA potentially reduces fat accumulation in worms by regulating lipid metabolic pathways. Although *lipl-1* was screened as a candidate gene using RT-qPCR (Figure 5), *lipl-1*, *lipl-2*, and *lipl-4* mutants, which belong to the same family, did not affect the fat accumulation-reducing effect of LPTOA (Figure 6a–c). Consequently, the results of this study refuted our hypotheses. In starved *C. elegans*, the expression of many genes involved in fatty acid degradation, including the *lipl* family, is markedly upregulated [52]. *lipl-1*, *lipl-2*, and *lipl-4* are involved in lipid metabolic pathways and are rapidly and transiently expressed in the intestine during fasting [53,54,55], suggesting that LPTOA-mediated fat accumulation reduction is not regulated by them. In contrast, considering that *lipl-1* expression is upregulated in response to reduced fat accumulation, LPTOA-mediated reduction in fat accumulation may induce a physiological response similar to that observed during starvation. Excessive fat loss in the body may trigger a starvation-like response considering that fat also serves as a crucial reservoir of nutrients. In conclusion, to clarify the mechanism by which a substance reduces fat accumulation, it is necessary to carefully distinguish between the direct effects of the substance and the reactions induced by the subsequent reduction in fat accumulation.

In contrast, LPTOA-mediated reduction in fat accumulation was suppressed in *pept-1* mutants (Figure 7a). The *pept-1* mutant exhibits increased fat accumulation and alterations in lipid composition as part of its phenotype [17,47]. PEPT-1, a dipeptide transporter, functions as an electrogenic proton-coupled symporter, with dipeptide absorption depending on the membrane potential and extracellular pH, to use the electrochemical proton gradient as its energy source [17,56]. Moreover, increased fat accumulation in the *pept-1* mutant is driven by the accelerated absorption of free fatty acids in the intestine, which is caused by decreased intestinal proton influx [17]. Therefore, two possible mechanisms are considered for the LPTOA-mediated reduction in fat accumulation in worms. The first possibility is that an unknown factor in LPTOA regulates intestinal proton influx. However, since RNAi of *pept-1* is known to reduce fat accumulation in worms [57], the role of *pept-1* in fat accumulation needs to be carefully investigated. Secondly, certain dipeptides in LPTOA may reduce fat accumulation in worms via PEPT-1. Certain dipeptides are known to extend the lifespan and suppress age-related stress in *C. elegans* [58] or alleviate diet-induced metabolism-associated fatty liver disease in mice [59]. Future studies should investigate the *C. elegans* genes and LPTOA substances involved in LPTOA-mediated reduction in fat accumulation.

Although previous studies have reported increased fat accumulation in the *pept-1* mutant used in this study, we did not observe a significant difference in baseline of fat accumulation levels compared to the wild-type. A possible explanation is the difference in the reagents used for observed fat accumulation. Previous studies utilized Sudan Black B, Nile Red, and BODIPY-C12 fatty acid to observe fat accumulation, whereas we used Oil Red O [17,47]. Sudan Black B and Oil Red O are fixative-based dyes. On the other hand, Nile Red and BODIPY are vital dyes used for observing fat accumulation in live worms [60]. Staining results from Nile Red and BODIPY do not always align with those of Sudan Black or Oil Red O, and they are known to sometimes overestimate fat accumulation [60]. Additionally, Oil Red O is well suited for staining neutral lipids, whereas Sudan Black has strong affinity for a variety of lipids, not only neutral lipids but also phospholipids and glycolipids [61]. Therefore, the experimental system we employed primarily evaluates neutral lipids, and the neutral lipid levels in *pept-1* mutants are suggested to be comparable to those in the wild-type.

To summarize our study, a pathway map was constructed (Figure 7b). Our study revealed that LPTOA does not directly influence dietary lipids in the intestinal tract of worms but reduces the accumulated fat in worms via *pept-1*. In addition, as the safety of LPTOA was demonstrated through a subacute toxicity study in mice (Figure 1a), LPTOA has been shown to possess both mammalian safety and fat-reducing effects through specific target gene (*pept-1*) in *C. elegans*. Thus, LPTOA may have potential applications as a probiotic for managing obesity in food and supplement products. However, significant differences exist between *C. elegans* and other mammals. For example, worms lack adipocytes and store lipids as lipid droplets in the intestine and hypodermis. Additionally, worms possess a simple intestine but lack blood vessels and organs such as the liver and pancreas. Therefore, to administer LPTOA to humans and companion animals, it is necessary to investigate whether its anti-obesity effects can be observed in various animals.

## Data Availability

The original contributions of this study are included in the article/Supplementary Materials. Further inquiries can be directed to the corresponding authors.

## Supplementary Materials

The following supporting information can be downloaded at https://www.mdpi.com/article/10.3390/microorganisms14030522/s1, Figure S1: Time course of lipase inhibition by each test substance; Table S1: Primer sequence; Table S2: Body weight changes and anatomical observations in mice subjected to subacute toxicity; Table S3: RT-qPCR data.
