# Supplementary material for: Lactiplantibacillus plantarum TO-A Reduces Fat Accumulation in Caenorhabditis elegans via pept-1
Source: Microorganisms. 2026 Feb 24;14(3):522. doi: 10.3390/microorganisms14030522 (PMC13028613; doi:10.3390/microorganisms14030522)
Supplement: Supplementary file 1 [file microorganisms-14-00522-s001.zip › Supplementary_Figure_TO-A.pdf]

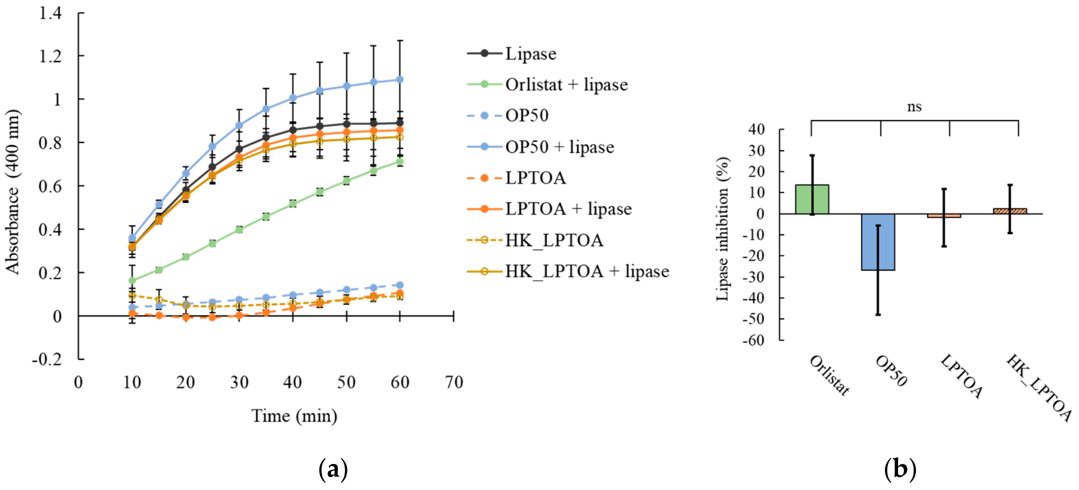

Figure S1. Time course of lipase inhibition by each test substance. Percentage lipase inhibition by orlistat, OP50, LPTOA, or HK_LPTOA was measured every 5 min from 10 to 60 min after substrate addition (a), with the end of the reaction at 60 min summarized separately (b). Each bar represents the standard error. Significant differences ($p > 0.05$) were determined using the Dunn's test (Holm) ($n = 3$). ns: non-significant.
